# Reducing 30-Day All-Cause Acute Exacerbation of Chronic Obstructive Pulmonary Disease Readmission Rate With a Multidisciplinary Quality Improvement Project

**DOI:** 10.7759/cureus.19917

**Published:** 2021-11-26

**Authors:** Jonathan Rohde, Abraham Joseph, Binta Tambedou, Nitesh K Jain, Syed Anjum Khan, Salim Surani, Rahul Kashyap, Thoyaja Koritala

**Affiliations:** 1 Hospital Medicine, Mayo Clinic, Mankato, USA; 2 Critical Care Medicine, Mayo Clinic, Mankato, USA; 3 Pulmonary and Critical Care Medicine, Texas A&M University, College Station, USA; 4 Medicine, University of North Texas Dallas, Dallas, USA; 5 Internal Medicine, Pulmonary Associates of Corpus Christi, Corpus Christi, USA; 6 Clinical Medicine, University of Houston, Houston, USA; 7 Anesthesiology and Critical Care, Mayo Clinic, Rochester, USA

**Keywords:** copd, quality improvement, health care quality improvement, evidence based medicine, chronic disease management, checklist

## Abstract

Our objective was to implement a comprehensive quality improvement project to decrease the 30-day readmission rate for all-cause acute exacerbation of chronic obstructive pulmonary disease (AECOPD) at a rural Midwestern community hospital in the United States. Prospective data were collected from January 1 to December 31, 2017. A total of 77 patients met the study criteria and were included for analysis. Baseline data analysis involved data for 72 patients from September 1, 2015, to October 1, 2016, and showed a 30.6% all-cause 30-day AECOPD readmission rate. The Define, Measure, Analyze, Improve, and Control (DMAIC) model was used for this quality improvement project. All aspects of this project were successfully implemented, and the resulting 30-day all-cause AECOPD readmission rate decreased to 16.9% during the study time frame. Through this comprehensive quality improvement project, the 30-day all-cause AECOPD readmission rate was reduced by 23.7%.

## Introduction

Chronic obstructive pulmonary disease (COPD) affects more than 11 million Americans and is the third leading cause of death in the United States (US) [1]. The cost of COPD was $32.1 billion in 2010 and was projected to cross $50 billion in the next decade [2]. In response to a high percentage of patients returning to the hospital within 30 days after discharge, which results in increased financial costs to the health care system, the Centers for Medicare & Medicaid Services (CMS) developed the Hospital Readmissions Reduction Program [3,4]. This program uses 30-day readmissions as a benchmark for evaluation of the care provided by organizations, and CMS financially penalizes organizations for excess 30-day readmissions. In 2015, CMS added acute exacerbation of COPD (AECOPD) to the list of diagnoses monitored [3,5]. The national 30-day all-cause readmission rate for AECOPD is approximately 20% [6].

A 57-bed rural community hospital in the Midwest had high AECOPD 30-day readmission rates for several years, and the hospital had accrued CMS penalties for 30-day readmissions since 2012. To reduce readmissions and avoid financial penalties, a multidisciplinary team designed and implemented a 12-month quality improvement (QI) project. The aim was to decrease the 30-day all-cause AECOPD readmission rate to less than the national rate of 20% by improving inpatient AECOPD treatment and ensuring continuity of care after discharge.

## Materials and methods

The Define, Measure, Analyze, Improve, and Control (DMAIC) model was used for this QI project. A retrospective chart review was performed to obtain the baseline 30-day all-cause AECOPD readmission rate. All available data were used for the chart review, which included the 13 months from September 1, 2015, to October 1, 2016. All data were used from the 13-month time frame to ensure the accuracy of readmission rates related to AECOPD. The use of 13 months of data also helped to account for seasonal factors that may affect hospital admissions throughout the year. Patients were included if they were older than 18 years and had a primary diagnosis of AECOPD on discharge. Patients were excluded if they were pursuing comfort care or hospice or were transferred to another hospital. Full patient data before September 1, 2015, were unavailable for analysis, but because the hospital had been penalized for 2012-2015, the team assumed that the baseline readmission rate calculated for the 13-month period in 2015 and 2016 would be like the rate from previous years.

A hospitalist nurse practitioner independently reviewed all included charts to ensure data accuracy. Because a patient’s diagnosis may change during hospitalization, the team chose to include only patients who had a documented discharge diagnosis of AECOPD to ensure data consistency without variability. All patients included in the baseline retrospective sample had a primary diagnosis of AECOPD documented on discharge. All patients included in the prospective sample also had a primary diagnosis of AECOPD on discharge. This method ensured the accuracy of the patient data without the need for interrater agreement.

A total of 72 patients were included in the baseline sample; 22 were readmitted within 30 days after discharge (30-day all-cause AECOPD readmission rate, 30.6%). Further analysis was performed to identify the major cause of readmission [4], and a subgroup was identified that was readmitted for additional treatment of AECOPD. Of the 22 patients readmitted, over half (12 patients; 54.5%) returned for further treatment of AECOPD.

Given that most patients who were readmitted needed further treatment of AECOPD, the team noted that the greatest opportunity for improvement involved the initial in-hospital treatment of AECOPD and the continuity of care after discharge. We assumed that the initial treatment was suboptimal, leading to discharge before the acute exacerbation had been fully managed and that the continuity of care after discharge was lacking, resulting in an inability for the care team to intervene soon enough after discharge to prevent readmissions [7].

**Figure 1:**
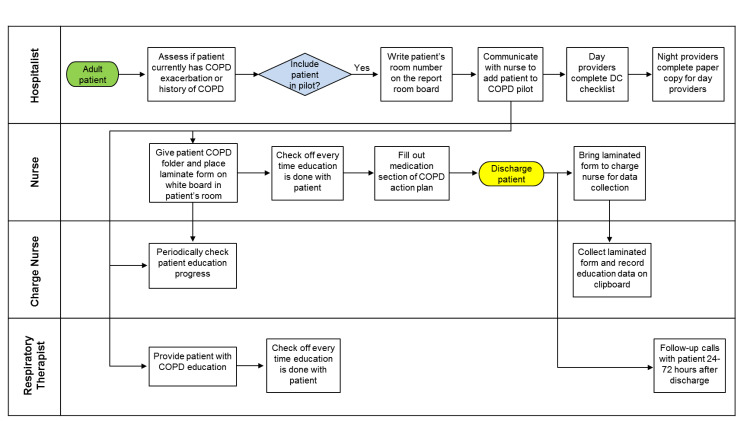
Process flow of quality improvement project COPD: chronic obstructive pulmonary disease; DC: discharge

A multidisciplinary team was assembled (a hospitalist, a nurse manager, a respiratory therapy manager, and a QI consultant). This team reviewed the literature and evidence-based guidelines to identify interventions to improve the treatment of AECOPD (Figure 1). The implementation team chose four initiatives: a COPD treatment checklist (see Appendix), an action plan, standardized education, and a follow-up telephone call within 72 hours after discharge [8]. Multiple sectors, including the airline industry, have shown that the use of checklists is extremely beneficial to ensure compliance. The team decided to introduce a checklist of key elements related to the inpatient and outpatient management of AECOPD to ensure that all aspects of care were addressed. Inpatient and outpatient management features were added to the checklist to ensure continuity from inpatient care to outpatient care after discharge.

To ensure compliance, goals for completion were determined for each initiative. The completion goals were 100% for the COPD treatment checklist, 100% for action plan initiation, 90% for patient education provided by nursing and respiratory therapy personnel, and 100% for follow-up telephone calls.

The prospective data included patients between January 1 and December 31, 2017. These 12 months of implementation were a counterpart to the 13 months of retrospective chart review for comparison of data. The inclusion and exclusion criteria used for the prospective patient data were the same as those for the baseline analysis. This project did not require additional staffing because the small amount of extra work was included in the duties of the current staff. p values less than 0.05 were considered statistically significant.

According to the policy activities that constitute research at our institution, this work met criteria for operational improvement activities exempt from the ethics review.

We defined the problem as AECOPD readmission reduction and measured the retrospective data to analyze the root causes. We subsequently identified several areas for improvement and designed the implementation plan with four initiatives: COPD treatment checklist, action plan, standardized education, and follow-up telephone call within 72 hours after discharge. We measured the impact of the QI initiatives in the prospective data and showed a statistically significant reduction in 30-day readmissions, and this reduction was sustained for over a year.

## Results

Baseline retrospective data included 72 patients, and 22 of them (30.6%) had an all-cause 30-day AECOPD readmission. Of the 22 patients readmitted, 12 (54.5%) were readmitted for further treatment of AECOPD. The prospective data included 77 patients, and 13 of them (16.9%) had an all-cause 30-day AECOPD readmission (Table 1). The decrease in readmission rates (from 30.6% to 16.9%) was statistically significant (P=.02). Of the 13 readmitted patients in the prospective data group, only four (30.8%) returned to the hospital for further treatment of AECOPD, which is a 23.7% reduction compared with the retrospective group (54.5%).

**Table 1:** Characteristics of patients in the retrospective data group and the prospective data group a Unless specified otherwise, values are number of patients (percentage of sample); b The retrospective data group consisted of patients in the study from September 1, 2015, to October 1, 2016; c The prospective data group consisted of patients in the study from January 1 through December 31, 2017. COPD: chronic obstructive pulmonary disease; y: years; d: days

Characteristic ^a^	Retrospective Data ^b^	Prospective Data ^c^
Total admissions, No.	72	77
Total readmissions	22 (30.6)	13 (16.9)
Readmissions due to COPD	12 (54.5)	4 (30.8)
Age of patients, y		
All patients		
Mean (SD)	73 (12.38)	72 (12.25)
Median	76	72
Patients not readmitted		
Mean (SD)	72 (12.42)	74 (12.40)
Median	76	70
Patients readmitted		
Mean (SD)	74 (12.49)	77 (11.11)
Median	77	81
Length of stay, d		
All patients		
Mean (SD)	3.68 (2.64)	4.58 (1.87)
Median	3	4
Patients not readmitted		
Mean (SD)	3.32 (2.09)	4.53 (1.89)
Median	3	4
Patients readmitted		
Mean (SD)	4.50 (3.50)	4.85 (1.77)
Median	3	4
Sex		
Male, all patients	35 (48.6)	40 (51.9)
Male, patients readmitted	12 (54.5)	8 (61.5)
Female, all patients	37 (51.4)	37 (48.0)
Female, patients readmitted	10 (45.5)	5 (38.5)
PFT for diagnosis		
All patients	34 (47.2)	29 (37.7)
Patients not readmitted	25 (50.0)	25 (39.1)
Patients readmitted	9 (40.9)	4 (30.8)
Follow-up clinic visit after admission		
All patients	48 (66.7)	51 (66.2)
Patients not readmitted	37 (74.0)	43 (67.2)
Patients readmitted	11 (50.0)	8 (61.5)
Patients with HF diagnosis		
All patients	37 (51.4)	44 (57.1)
Patients not readmitted	27 (54.0)	34 (53.1)
Patients readmitted	10 (45.5)	10 (76.9)
Active smoking		
All patients	21 (29.2)	23 (29.9)
Patients not readmitted	15 (30.0)	20 (31.3)
Patients readmitted	6 (27.3)	3 (23.1)
Supplemental oxygen at discharge		
All patients		41 (53.2)
Patients not readmitted		35 (54.7)
Patients readmitted		6 (46.2)
New medication at discharge		
All patients		61 (79.2)
Patients not readmitted		50 (78.1)
Patients readmitted		11 (84.6)
Comorbidities		
All patients		68 (88.3)
Patients not readmitted		56 (87.5)
Patients readmitted		12 (92.3)
PFT ordered at discharge		
All patients		40 (51.9)
Patients not readmitted		35 (54.7)
Patients readmitted		5 (38.5)

In the retrospective data group, the mean (SD) age of all patients was 73 (12.38) years (median, 76 years), and the mean age of those readmitted was 74 (12.49) years (median, 77 years). In the prospective data group, the mean age of all patients was 72 (12.25) years (median, 72 years), and the mean age of those readmitted was 77 (11.11) years (median, 81 years). In the retrospective data group, the mean length of stay (LOS) for all patients was 3.68 (2.64) days (median, 3 days); the mean LOS for those readmitted was 4.50 (3.50) days (median, 3 days); and the mean LOS for those not readmitted was 3.32 (2.09) days (median, 3 days). In the prospective data group, the mean LOS for all patients was 4.58 (1.87) days (median, 4 days); the mean LOS for those readmitted was 4.85 (1.77) days (median, 4 days); and the mean LOS for those not readmitted was 4.53 (1.89) days (median, 4 days).

The total number of men was 35 (48.6%) in the retrospective data group and 40 (51.9%) in the prospective data group. The number of men readmitted was 12 (54.5%) in the retrospective data group and eight (61.5%) in the prospective data group. The total number of women was 37 (51.4%) in the retrospective data group and 37 (48.0%) in the prospective data group. The number of women readmitted was 10 (45.5%) in the retrospective group and five (38.5%) in the prospective group.

Pulmonary function testing (PFT) documented evidence of COPD in 34 of the 72 patients (47.2%) in the retrospective data group and in 29 of the 77 patients (37.7%) in the prospective data group. Of the 50 patients in the retrospective data group who were not readmitted, 25 (50%) had evidence of COPD; of the 64 in the prospective data group who were not readmitted, 25 (39.1%) had evidence of COPD. Of the patients who had evidence of COPD and were readmitted, nine of 22 (40.9%) were in the retrospective data group and four of 13 (30.8%) were in the prospective data group. Of all the patients who had a follow-up clinic visit after admission, 48 of 72 (66.7%) were in the retrospective data group and 51 of 77 (66.2%) were in the prospective data group. Of all the patients who had a follow-up clinic visit but were not readmitted, 37 of 50 (74%) were in the retrospective data group and 43 of 64 (67.2%) were in the prospective data group. Of all the patients who had a follow-up clinic visit and were readmitted, 11 of 22 (50.0%) were in the retrospective data group and eight of 13 (61.5%) were in the prospective data group.

Of all the patients with a diagnosis of heart failure (HF), 37 of 72 (51.4%) were in the baseline retrospective data group and 44 of 77 (57.1%) were in the baseline prospective data group. Of all the patients with a diagnosis of HF who were not readmitted, 27 of 50 (54.0%) were in the retrospective data group and 34 of 64 (53.1%) were in the prospective data group. Of all the patients with a diagnosis of HF who were readmitted, 10 of 22 (45.5%) were in the retrospective data group and 10 of 13 (76.9%) were in the prospective data group. Of all the patients who were actively smoking, 21 of 72 (29.2%) were in the retrospective data group and 23 of 77 (29.9%) were in the prospective data group. Of the patients who were not readmitted and were actively smoking, 15 of 50 (30%) were in the retrospective data group and 20 of 64 (31.3%) were in the prospective data group. Of the patients who were readmitted and were actively smoking, six of 22 (27.3%) were in the retrospective data group and three of 13 (23.1%) were in the prospective data group.

Several changes were implemented and tracked, as additional data points (Table 1), for the prospective data group to identify other potential causes leading to AECOPD readmissions. These included addressing supplemental oxygen needs at discharge, education regarding new medications on discharge for the treatment of COPD, identifying and addressing other medical comorbidities as applicable, and ordering PFTs on discharge to assess COPD status.

Adherence to the initiatives was tracked throughout the study. Hospitalists completed the COPD treatment checklist and action plan initiation for 96% of patients. Standardized education during hospitalization was completed twice by the nursing staff for 81% of the patients and twice by respiratory therapists for 92% of the patients. A follow-up telephone call was completed for 94% of the patients.

## Discussion

The retrospective chart review showed a 30-day all-cause AECOPD readmission rate of 30.6%. Although patients were readmitted to the hospital within 30 days after discharge for several reasons, the main reason was the need for ongoing treatment of AECOPD, which most likely reflected inadequate initial treatment before hospital discharge or a lack of continuity of care after discharge. The QI project implemented several initiatives to address this problem.

After the 12-month QI project, the all-cause 30-day AECOPD readmission rate decreased to 16.9%. This decrease resulted from improvement in care provided by the multidisciplinary team. The initiatives were successful, which is evident by the reduction of the total number of readmissions and by the decrease in the number of patients readmitted for further treatment of AECOPD. Our retrospective review found a readmission rate of 50% in 30 days among patients who were admitted for COPD exacerbation. After the project was implemented, readmission for AECOPD decreased from 54.5% to 30.8%, which is a clinically significant decrease of 23.7%. This reduction shows that a comprehensive multidisciplinary QI project decreases 30-day readmission rates for this subgroup and for the overall AECOPD patient population. Improvement in the patient's overall care, including care provided through multiple disciplines, directly corresponded to improvement in 30-day AECOPD readmission outcomes.

To improve the medical treatment of AECOPD, a COPD treatment checklist was implemented. Checklists are effective tools for ensuring that protocols are followed consistently and completely, and they have been implemented inside and outside the medical field. Our team modified the Global Initiative for Chronic Obstructive Lung Disease (GOLD) guidelines to design a unique checklist that could be used to ensure that inpatient management of AECOPD patients was based on the gold standard of care and to ensure continuity of care after discharge [9]. Completing the checklist ensured that the hospitalist followed all recommended guidelines for the inpatient treatment of AECOPD and addressed the outpatient factors. The checklist was started on the day of admission and completed by the day of discharge. Completion was documented in the hospital summary, which also served as a form of communication to the outpatient primary care provider to ensure continuity of care [10]. Most COPD patients admitted to the hospital had no PFT results documented, their vaccinations were outdated, or they were receiving inadequate medications according to their GOLD class. The checklist addressed these areas so that the outpatient primary care provider can take the action during follow-up visits. The COPD treatment checklist (completion rate, 96%) helped ensure that every patient received standardized, evidence-based care.

When nursing and respiratory therapy patient education was analyzed before implementation of the project, the team identified a major gap. Before implementation, standardized COPD education was not provided. The nursing staff was uncomfortable with educating the patients, and the appropriate number of education sessions before discharge had not been determined. The respiratory therapists were educating patients, but the type of education and the number of sessions were unknown.

To improve education, standardized education material was implemented, and the team decided that the patients would be educated four times during hospitalization. The nurses were responsible for two education sessions, and the respiratory therapists were responsible for the other two sessions. To ensure adequate patient understanding, the teach-back method was implemented [11]. Compliance with the number of times education was performed was monitored through frequent audits of the medical charts. The overall project education compliance was 81% for the nursing staff and 92% for the respiratory therapists.

After hospitalization, transition back to the community can be difficult for patients, especially in the first few days after discharge. The team decided to implement a follow-up telephone call within 72 hours after discharge. The telephone call was performed by the inpatient respiratory therapists to provide continuity of care [10] because the inpatient respiratory therapists already had a rapport with the patients and an understanding of the patients’ individual needs. The implementation team designed a telephone questionnaire to evaluate how patients had transitioned back to their home situations and inquire about how they were doing after discharge from the hospital. The respiratory therapists could clarify information if patients were confused about management or medication use. The telephone call also provided an opportunity for the respiratory therapist to evaluate the condition of the patient after discharge. If any concerns were identified, the respiratory therapist reviewed the action plan and helped the patient schedule a recheck evaluation in the clinic if needed. Telephone calls after discharge were documented in the electronic health record, and compliance was monitored through frequent chart audits. The overall telephone call compliance was 94% for the project.

One of the greatest project challenges was that compliance did not approach 100% until approximately four months after the project was implemented. The initiatives were new for the hospital team and involved several disciplines. A key factor in boosting compliance was encouraging accountability through audits. An email sent to all staff members every other week communicated compliance with the initiatives. When the whole team understood where compliance needed to be increased, colleagues could help each other be accountable for the initiatives.

Limitations

All available patient data were used for the retrospective results, but these data were from only September 2015 to October 2016, which was a limitation for identifying the year with the greatest number of readmissions between 2012 and 2016. The team assumed that the readmission rate for the years before 2015 correlated with the readmission rate identified from the retrospective data, since the hospital had accrued penalties from high AECOPD readmissions from 2012 to 2015. In addition, the initiative that had the greatest effect in reducing the readmission rate is unknown because it was not possible to quantify the contribution of each initiative. In future projects, comparison between a control group and an intervention group could allow identification of the most beneficial initiative. Although the initiatives involved extra work for the staff, this increase in work was offset by improvement in outcomes, so that the staff adopted the initiatives as part of their work.

## Conclusions

This QI project improved the care of AECOPD patients and decreased the 30-day all-cause AECOPD readmission rate to less than the national rate. The QI project has been sustainable, and the initiatives implemented have become common practice at the institution. The 30-day all-cause AECOPD readmission rate has remained below the goal set forth at the beginning of the project. 30-day all-cause AECOPD readmission rate decreased to 16.9% as of December 2017. As of September 2018, even nine months after completion of the QI project, the 30-day all-cause AECOPD readmission rate continued to remain low at 16.4%. The initiatives used in this QI project have the potential to enhance care and should be explored by other institutions.

## Appendices

COPD Treatment Checklist

**Table 2:** COPD treatment checklist COPD: chronic obstructive pulmonary disease; PFT: pulmonary function test

Have the diagnosis and action plan education been done?
Has the action plan for COPD exacerbation been initiated?
Were initial PFTs done for diagnosis?
Were PFTs done within the past year? If not, were they ordered for after discharge?
Is the patient a current tobacco user? Is the patient willing to quit?
Was tobacco cessation prescribed on discharge?
Was pulmonary hygiene initiated in the hospital?
Are the patient’s vaccinations current (influenza, pneumonia, tetanus, diphtheria, and pertussis)?
Is the patient receiving oxygen? Does the patient need an oxygen titration study and a nocturnal oximetry recommendation after the acute disease resolves?
Has a dietitian been consulted to give guidance for a high-protein, high-calorie diet?
Does the patient need a sleep apnea study? (Is the patient obese?)
Does the patient adhere to the COPD medication regimen at home?
Did the patient receive new medications during hospitalization and appropriate inpatient education on how to use the new medication?
Were corticosteroids ordered?
Was a pulmonary rehabilitation evaluation recommended on discharge?
Have comorbid conditions been optimized?
Is a follow-up ordered for 3 to 5 days after discharge?
Does the patient need a palliative-care consultation because of end-stage COPD?
Was the patient enrolled in remote patient monitoring?

Author contributions

Jonathan E. Rohde: responsible for concept and design of the work and for data analysis and interpretation; undertook drafting and final approval of the manuscript; accountable for the work.

Abraham Joseph: responsible for data acquisition and undertook manuscript revision and final approval of the manuscript; accountable for the work.

Binta Tambedou: responsible for data acquisition and undertook manuscript revision and final approval of the manuscript; accountable for the work.

Nitesh Jain, Syed Anjum Khan, Salim Surani, and Rahul Kashyap: responsible for manuscript revision and final approval of the manuscript.

Thoyaja Koritala: responsible for data acquisition and undertook manuscript revision and final approval of the manuscript; accountable for the work.
